# Controlled Irrigation Mitigates Flooding-Induced Yield Loss in Rice (*Oryza sativa* L.) at the Jointing Stage by Regulating Growth and Biomass Allocation

**DOI:** 10.3390/plants15152405

**Published:** 2026-08-06

**Authors:** Yanmei Yu, Yujiang Xiong, Yan Meng, Peng Chen, Hang Guo

**Affiliations:** 1 School of Water Conservancy and Electric Power, Heilongjiang University, Harbin 150080, China; 2 Changjiang River Scientific Research Institute, Wuhan 430010, China; 3 Heilongjiang Province Hydraulic Research Institute, Harbin 150080, China; 4 College of Hydraulic Science and Engineering, Northeast Agricultural University, Harbin 150030, China

**Keywords:** water-saving irrigation, flooding stress, canopy regulation, photosynthetic capacity, dry matter accumulation, yield formation

## Abstract

Flooding during the rice (*Oryza sativa* L.) jointing stage threatens yield stability in regions with concentrated rainfall and limited drainage. However, the extent to which the water regime before flooding modifies rice growth and yield formation remains poorly understood. A pot experiment was conducted over two years to compare controlled irrigation (CI) with conventional flooding irrigation (CF) across three flooding depths and two durations imposed at the jointing stage. Flooding increased tiller number, plant height, and total leaf area, but reduced net photosynthetic rate and grain yield. Yield loss increased with flooding depth and duration. Under corresponding flooding treatments, CI moderated vegetative expansion and maintained a higher net photosynthetic rate, greater root dry matter, and a higher root-to-shoot ratio than CF. Relative to the corresponding non-flooded controls, yield loss ranged from 4.11% to 39.33% under CI and from 5.63% to 52.50% under CF. The lower yield loss under CI was associated with greater effective panicle number, higher seed setting rate, and better maintenance of photosynthetic activity and root biomass. These findings indicate that the water regime before flooding can influence biomass allocation and yield formation during the jointing stage flooding. Controlled irrigation combined with timely drainage may help reduce yield risk in rice systems exposed to temporary flooding.

## 1. Introduction

Rice (*Oryza sativa* L.) production plays a central role in global food security and remains highly reliant on water-intensive irrigation practices. Continuous flooding has long been adopted in paddy systems because it ensures a stable water supply and effective weed control. However, growing competition for freshwater resources and increasing climatic variability have placed significant constraints on this traditional practice [1,2]. Improving water use efficiency while maintaining yield stability has therefore become a central objective of rice water management [3,4,5]. Water-saving irrigation strategies, particularly alternate wetting and drying (AWD) and controlled irrigation (CI), have been widely developed to address this challenge. Numerous field studies and meta-analyses indicate that these approaches can substantially reduce irrigation water input while sustaining grain yield under appropriate management [1,2,6,7,8,9]. Unlike conventional flooding, CI regulates irrigation based on soil moisture thresholds rather than maintaining a persistent surface water layer, thereby reducing non-productive water losses and improving water productivity [10,11].

Despite these benefits, rice cultivation under water-saving irrigation remains vulnerable to short-term flooding events. In monsoonal regions, concentrated rainfall and limited drainage capacity frequently result in temporary flooding that can override irrigation management and expose rice plants to excess water stress, even under CI or AWD systems [12,13]. Flooding restricts oxygen diffusion in the root zone, disrupts photosynthesis and assimilate transport, and alters biomass distribution, often leading to yield loss [14,15]. Flooding damage is strongly influenced by flooding depth, duration, and the growth stage at which stress occurs [16,17]. Among rice developmental stages, the jointing stage represents a critical transition from vegetative growth to reproductive development, during which internode elongation and panicle differentiation proceed rapidly. Flooding during this stage has been shown to suppress tiller survival, reduce photosynthetic capacity, and impair panicle formation, resulting in irreversible yield penalties [16,17]. Experimental evidence further suggests that deeper and longer flooding events intensify these effects and limit recovery following stress [18,19].

Most previous studies on flooding stress in rice have focused on conventional flooding irrigation systems, where background water levels are already high. Under such conditions, additional flooding primarily intensifies anaerobic stress. In contrast, rice grown under CI experiences distinct soil water regimes prior to flooding, characterized by reduced surface ponding and periodic soil aeration. These conditions prior to flooding may alter growth patterns and biomass allocation, potentially influencing plant responses to subsequent flooding stress [20]. However, whether CI can mitigate yield loss by flooding at sensitive stages such as the jointing stage remains insufficiently understood. Recent studies have emphasized that yield responses to water stress are closely associated with coordinated regulation of canopy development, carbon assimilation, and biomass allocation, rather than with individual growth traits alone [21,22,23,24]. Under water-saving irrigation, moderated vegetative growth and improved coordination between carbon sources and sinks have been linked to yield stability, whereas excessive flooding may disrupt these regulatory processes [25,26]. Integrating physiological traits, biomass allocation, and yield components is therefore essential for understanding rice performance under combined irrigation and flooding stresses.

Although the individual effects of irrigation regime and flooding stress have been widely examined, their interactive effects under controlled irrigation at the jointing stage remain poorly quantified. This knowledge gap is particularly relevant for rice production systems in regions where water resources are limited but rainfall is intensive. Accordingly, this study aimed to (i) quantify the effects of flooding depth and duration at the jointing stage on rice growth, physiology, biomass allocation, and yield; (ii) compare rice responses to flooding stress under controlled irrigation and conventional flooding irrigation; and (iii) clarify the physiological and biomass allocation responses through which irrigation management influences rice responses to flooding.

## 2. Results

### 2.1. Controlled Irrigation Constrained Flooding-Induced Vegetative Overgrowth

**Figure 1:**
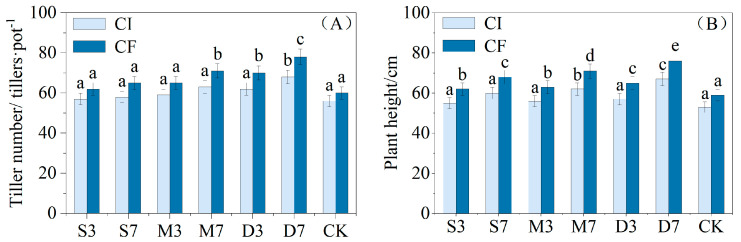
Effects of flooding stress on tiller number (**A**) and plant height (**B**) under controlled irrigation (CI) and conventional flooding irrigation (CF). Tiller number and plant height were measured during the jointing stage on the first day after flooding termination. S, M, and D denote flooding depths of 1/3, 2/3, and full plant height, respectively; 3 and 7 denote flooding durations of 3 and 7 d, respectively; CK denotes the non-flooded control. Values are means ± SE (n = 6, three replicates per year pooled across 2023 and 2024). Different lowercase letters indicate significant differences among treatment combinations according to one-way ANOVA followed by Tukey’s HSD test at *p* < 0.05. One-way ANOVA showed significant treatment combination effects on tiller number (F (13, 70) = 9.40, *p* < 0.05) and plant height (F (13, 70) = 11.96, *p* < 0.05).

Flooding imposed at the jointing stage significantly altered rice vegetative growth, as reflected by changes in tiller number and plant height, with clear contrasts between irrigation regimes (Figure 1). Relative to the non-flooded control, all flooding treatments increased both tiller number and plant height under CI and CF, indicating that jointing stage flooding generally promoted vegetative growth. Under CF, flooding induced a pronounced increase in tiller number across all flooding depths and durations. The magnitude of tiller proliferation increased progressively with flooding severity, particularly under longer flooding durations. In contrast, CI consistently moderated this response. Under comparable flooding conditions, tiller number under CI remained significantly lower than under CF, with the difference becoming more evident under moderate and severe flooding treatments.

Plant height exhibited a similar but more depth dependent response. Flooding significantly increased plant height under both irrigation regimes, and height elongation intensified with increasing flooding depth and duration. Under CF, plant height increased markedly across all flooding treatments, whereas CI consistently limited the extent of elongation. Across all flooding scenarios, plants grown under CI were significantly shorter than those under CF, with the largest differences observed under severe flooding (*p* < 0.05). Overall, flooding at the jointing stage stimulated tiller proliferation and stem elongation, while controlled irrigation effectively constrained excessive vegetative growth under flooding stress, resulting in a distinctly moderated growth pattern compared with conventional flooding irrigation.

### 2.2. Controlled Irrigation Moderated Canopy Expansion and Sustained Photosynthetic Capacity

**Figure 2:**
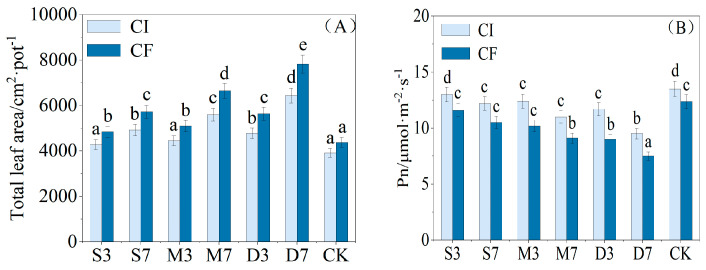
Effects of flooding stress on total leaf area (TLA, (**A**)) and net photosynthetic rate (Pn, (**B**)) under controlled irrigation (CI) and conventional flooding irrigation (CF). Total leaf area and Pn were measured during the jointing stage on the first day after flooding termination. S, M, and D denote flooding depths of 1/3, 2/3, and full plant height, respectively; 3 and 7 denote flooding durations of 3 and 7 d, respectively; CK denotes the non-flooded control. Values are means ± SE (n = 6, three replicates per year pooled across 2023 and 2024). Different lowercase letters indicate significant differences among treatment combinations according to one-way ANOVA followed by Tukey’s HSD test at *p* < 0.05. One-way ANOVA showed significant treatment combination effects on total leaf area (F (13, 70) = 34.47, *p* < 0.05) and net photosynthetic rate (F (13, 70) = 23.30, *p* < 0.05).

Total leaf area and photosynthetic performance responded differently to flooding stress under the two irrigation regimes (Figure 2). Compared with the non-flooded control, flooding increased total leaf area at the jointing stage under both CI and CF, with larger increases observed as flooding depth and duration increased. On the first day after flooding termination, total leaf area increased by 9.20–64.42% under CI relative to CI-CK and by 10.68–78.63% under CF relative to CF-CK. The largest increase was observed under D7, reaching 64.42% under CI and 78.63% under CF. Under CF, total leaf area increased substantially across most flooding treatments, particularly under moderate and severe flooding. In contrast, total leaf area under CI remained consistently lower than under CF under comparable flooding conditions, indicating that controlled irrigation moderated flooding-induced leaf area expansion.

In contrast to total leaf area, net photosynthetic rate (Pn) declined under flooding stress relative to the non-flooded control for both irrigation regimes. Across flooding treatments, Pn decreased by 3.70–29.63% under CI relative to CI-CK and by 6.27–39.37% under CF relative to CF-CK. The greatest reduction occurred under D7, where Pn decreased by 29.63% under CI and 39.37% under CF. However, under all flooding treatments, CI maintained significantly higher Pn values than CF. Under the same flooding scenarios, Pn under CI was 12.13–30.00% higher than that under CF, indicating that controlled irrigation alleviated the flooding-induced decline in photosynthetic capacity. This advantage of CI in sustaining photosynthetic capacity was consistent across flooding depths and durations. Collectively, flooding at the jointing stage promoted leaf area expansion while suppressing photosynthetic capacity, whereas controlled irrigation moderated excessive leaf area development and sustained a more favorable canopy source function.

### 2.3. Controlled Irrigation Improved Biomass Accumulation and Allocation Under Flooding Stress

**Figure 3:**
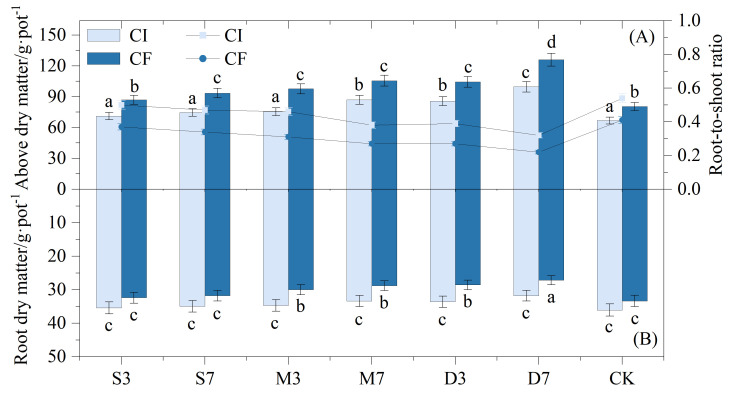

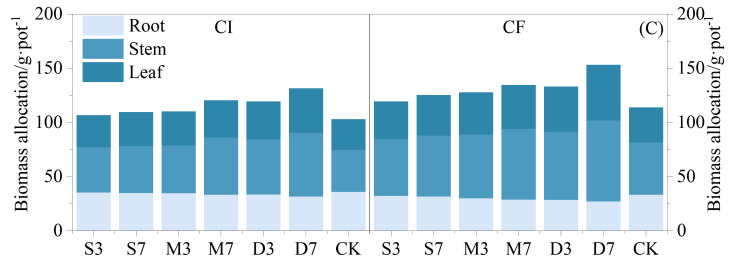
Effects of flooding stress on aboveground dry matter and root-to-shoot ratio (**A**), root dry matter (**B**), and biomass allocation characteristics (**C**) under controlled irrigation (CI) and conventional flooding irrigation (CF). Biomass accumulation and allocation were determined during the jointing stage on the first day after flooding termination. S, M, and D denote flooding depths of 1/3, 2/3, and full plant height, respectively; 3 and 7 denote flooding durations of 3 and 7 d, respectively; CK denotes the non-flooded control. Values are means ± SE (n = 6, three replicates per year pooled across 2023 and 2024). Different lowercase letters indicate significant differences among treatment combinations according to one-way ANOVA followed by Tukey’s HSD test at *p* < 0.05. One-way ANOVA showed significant treatment combination effects on aboveground dry matter (F (13, 70) = 14.68, *p* < 0.05) and root dry matter (F (13, 70) = 11.26, *p* < 0.05).

Flooding applied at the jointing stage led to clear changes in biomass accumulation, and these changes differed consistently between irrigation regimes (Figure 3). Under both CI and CF, aboveground dry matter increased as flooding depth and duration increased (Figure 3A). However, for any given flooding treatment, the increase in aboveground dry matter was always smaller under CI than under CF, indicating that controlled irrigation restrained the expansion of aboveground biomass under flooding stress. This difference was especially pronounced under the severe flooding treatments D7, where aboveground dry matter under CI was 20.95% lower than that under CF. Root dry matter responded differently from aboveground biomass (Figure 3B). Under CF, root dry matter decreased steadily with increasing flooding severity, with the largest reductions observed under deeper and longer flooding treatments. In contrast, root dry matter under CI showed much smaller variation across flooding treatments and remained significantly higher than that under CF at corresponding flooding levels (*p* < 0.05). Under D7, root dry matter under CI was 17.09% higher than that under CF, suggesting that controlled irrigation helped maintain belowground biomass under severe flooding stress.

The contrasting responses of aboveground and root biomass were reflected in biomass allocation patterns (Figure 3A). Across all flooding treatments, plants grown under CI maintained a consistently higher root-to-shoot ratio than those under CF. Moreover, the difference between irrigation regimes became more pronounced as flooding depth and duration increased. Under the severe flooding treatments D7, the root-to-shoot ratio under CI was 48.13% higher than that under CF. This indicates that controlled irrigation favored the retention of biomass in the belowground component under flooding stress. Taken together, flooding during the jointing period promoted biomass accumulation in aboveground tissues while constraining root growth. Controlled irrigation limited excessive accumulation of aboveground biomass and mitigated flooding-induced losses in root dry matter, thereby maintaining a more balanced pattern of biomass allocation under flooding conditions.

### 2.4. Controlled Irrigation Mitigated Flooding-Induced Yield Loss and Stabilized Yield Components

**Figure 4:**
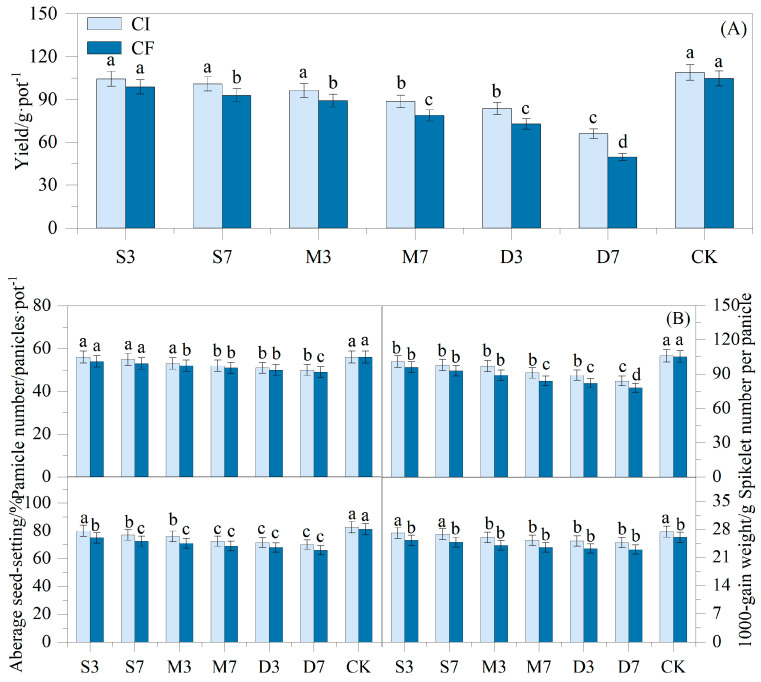
Effects of flooding stress on grain yield (**A**) and yield components (**B**) under controlled irrigation (CI) and conventional flooding irrigation (CF). Grain yield and yield components were determined at physiological maturity. S, M, and D denote flooding depths of 1/3, 2/3, and full plant height, respectively; 3 and 7 denote flooding durations of 3 and 7 d, respectively; CK denotes the non-flooded control. Yield components include effective panicle number, spikelet number per panicle, seed setting rate, and 1000-grain weight. Values are means ± SE (n = 6, three replicates per year pooled across 2023 and 2024). Different lowercase letters indicate significant differences among treatment combinations according to one-way ANOVA followed by Tukey’s HSD test at *p* < 0.05. One-way ANOVA showed significant treatment combination effects on grain yield (F (13, 70) = 69.56, *p* < 0.05), effective panicle number (F (13, 70) = 16.51, *p* < 0.05), spikelet number per panicle (F (13, 70) = 14.46, *p* < 0.05), seed setting rate (F (13, 70) = 5.01, *p* < 0.05), and 1000-grain weight (F (13, 70) = 2.96, *p* < 0.05).

Flooding imposed at the jointing stage significantly reduced grain yield, with pronounced differences between irrigation regimes (Figure 4A). Across all flooding treatments, grain yield under CI was consistently higher than under CF. For both irrigation regimes, grain yield declined progressively with increasing flooding depth and duration. Relative to the corresponding non-flooded controls, yield loss ranged from 4.11% to 39.33% under CI and from 5.63% to 52.50% under CF. Specifically, yield losses under S3, S7, M3, M7, D3, and D7 were 4.11, 7.33, 11.60, 18.64, 23.14, and 39.33% under CI, respectively, compared with 5.63, 11.28, 14.88, 24.74, 30.45, and 52.50% under CF. Yield differences between CI and CF were small under mild flooding but became increasingly evident under moderate and severe flooding. Under the severe flooding treatments D3 and D7, yield loss under CI was 7.31 and 13.17 percentage points lower than that under CF, respectively. The largest difference occurred under full submergence for 7 days, where grain yield under CI was significantly higher than under CF (*p* < 0.05). No significant yield difference was observed between irrigation regimes in the non-flooded control.

Analysis of yield components revealed that effective panicle number and seed setting rate were more sensitive to flooding stress than spikelet number per panicle and 1000-grain weight (Figure 4B). With increasing flooding severity, effective panicle number and seed setting rate declined under both irrigation regimes, but remained consistently higher under CI. In contrast, spikelet number per panicle and 1000-grain weight exhibited relatively small variations among flooding treatments and between irrigation regimes. Overall, flooding-induced yield loss increased with flooding severity and duration, whereas controlled irrigation consistently mitigated yield reductions. The higher grain yield observed under CI was associated with greater effective panicle number and seed setting rate.

### 2.5. Correlation Analysis of Growth Traits, Biomass Allocation, and Grain Yield

**Figure 5:**
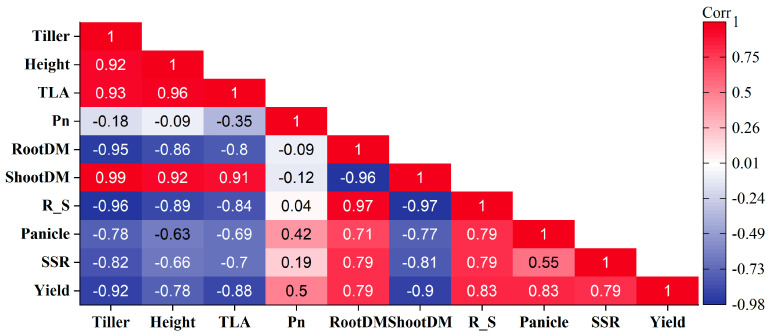
Correlation matrix illustrating relationships among growth traits, biomass allocation variables, and grain yield across treatments. Red and blue colors indicate positive and negative correlations, respectively. Correlation coefficients are based on pooled data from 2023 and 2024.

Correlation analysis revealed coherent relationships among growth traits, biomass allocation, and grain yield across treatments (Figure 5). Grain yield was negatively correlated with tiller number, plant height, and total leaf area, whereas positive correlations were observed between grain yield and net photosynthetic rate, root dry matter, root-to-shoot ratio, effective panicle number, and seed setting rate. Among all variables, the root-to-shoot ratio and effective panicle number exhibited the strongest positive associations with grain yield, while indicators of excessive vegetative growth showed consistent negative correlations. These relationships suggest that restrained vegetative growth combined with optimized biomass allocation was associated with improved yield performance under flooding stress.

## 3. Discussion

### 3.1. Controlled Irrigation Moderated Vegetative Responses to Flooding at the Jointing Stage

The contrasting responses under CI and CF indicate that the water regime before flooding influenced the subsequent morphological adjustment of rice. The increase in plant height was consistent with the escape strategy commonly observed under partial or complete submergence. Flooding restricts oxygen diffusion into the soil and induces root hypoxia. In rice, the resulting accumulation of ethylene, together with changes in gibberellin and abscisic acid signaling, can promote internode and leaf sheath elongation, enabling shoots to approach or emerge above the water surface [27,28]. Aerenchyma formation and adventitious root development may further improve internal oxygen transport under submerged conditions [27]. These responses can support short-term survival, but rapid elongation also requires substantial energy and carbohydrate remobilization. When flooding persists, depletion of carbohydrate reserves may weaken subsequent recovery [17,27,28]. Thus, the greater plant height observed under flooding primarily reflects morphological acclimation to oxygen deficiency rather than an overall improvement in plant growth.

The increase in tiller number requires consideration of the developmental stage. Rice responses to flooding vary with the timing, depth, and duration of stress [14,16,17,18]. Short periods of submergence during tillering may maintain or even stimulate vegetative development under certain conditions [18], whereas flooding from jointing to booting generally reduces tiller survival and interferes with panicle development [16,17]. In the present experiment, flooding occurred during the transition from late tillering to jointing. The greater tiller number under CF may therefore have resulted from the emergence or temporary retention of late tillers under abundant water conditions. However, effective panicle number declined as flooding became more severe, showing that the additional tillers did not necessarily develop into productive panicles. Excessive late tillering may also intensify competition for assimilates between vegetative organs and developing reproductive sinks [16,17].

Compared with CF, CI produced smaller increases in plant height, tiller number, and total leaf area, suggesting a more moderate morphological response to flooding. Unlike continuous flooding, CI maintained no persistent surface water layer before treatment and allowed periodic soil aeration. Such conditions can alter root activity and plant responses to subsequent water stress [20,21]. The preconditioning effect of CI may therefore have limited excessive shoot elongation and late tiller development after flooding. More restrained vegetative growth could reduce assimilate consumption by nonproductive organs and support a better coordinated transition from vegetative to reproductive development.

### 3.2. Controlled Irrigation Improved Canopy Function and Biomass Allocation

Flooding increased total leaf area but progressively reduced net photosynthetic rate, indicating that canopy expansion did not produce a corresponding improvement in carbon assimilation. Compared with CF, CI maintained a higher net photosynthetic rate together with a more moderate leaf area. Its advantage was therefore associated mainly with the preservation of canopy function rather than greater leaf production. Flooding restricts oxygen diffusion in the root zone, reduces energy supply and root water uptake, and subsequently disrupts leaf water status, stomatal regulation, and photosynthetic carbon assimilation [14,15,19,27]. Excessive canopy expansion may also increase mutual shading and respiratory consumption, thereby limiting the contribution of newly formed leaves to whole plant carbon gain [16,19]. The combination of sustained photosynthetic activity and restrained leaf area expansion under CI consequently indicates a more effective canopy source during recovery.

Differences in root biomass further distinguished the responses to the two irrigation regimes. Oxygen deficiency restricts aerobic respiration and energy production in roots, leading to reduced root activity, nutrient acquisition, and biomass accumulation [14,15,27]. In the present study, CI maintained greater root dry matter and a higher root-to-shoot ratio than CF, particularly under severe flooding. This pattern agrees with previous evidence that water-saving irrigation with periodic soil aeration promotes root development and improves resource acquisition in rice [11,21]. The alternating soil water conditions before flooding may have established a stronger root system, allowing plants under CI to retain more belowground biomass after exposure to oxygen deficiency. By contrast, the continuously flooded conditions under CF favored shoot expansion but were associated with a greater decline in root biomass.

The higher net photosynthetic rate, moderate leaf area, greater root dry matter, and higher root-to-shoot ratio under CI together indicate closer coordination between canopy carbon assimilation and belowground biomass maintenance. A larger functional root system supports water and nutrient acquisition, while sustained leaf photosynthesis provides assimilates for tissue repair and subsequent reproductive development [15,19,21]. This coordinated allocation between shoots and roots likely contributed to the lower yield losses observed under CI, especially as flooding depth and duration increased.

### 3.3. Controlled Irrigation Stabilized Yield Components and Reduced Grain Yield Loss

Flooding at the jointing stage reduced grain yield under both irrigation regimes, consistent with previous studies that identified the jointing to booting period as a sensitive phase of rice yield formation [14,16,17]. During this transition, productive tiller survival, stem elongation, and panicle differentiation occur concurrently. Disturbances to these processes can have lasting consequences for reproductive development. Zhen et al. [16] reported that waterlogging during the jointing to booting stage restricted rice growth and yield formation, while Yu et al. [17] found that submergence weakened the recovery of rice after flooding and reduced final grain yield. The present results support these findings and further indicate that the water regime before flooding influenced the subsequent yield response.

Under corresponding flooding treatments, CI generally maintained higher effective panicle number, seed setting rate, and grain yield than CF. In comparison, spikelet number per panicle and 1000-grain weight varied less between the two irrigation regimes. Together with the positive correlations of grain yield with effective panicle number and seed setting rate, this pattern suggests that the yield advantage under CI was more closely associated with the maintenance of productive panicle formation and seed setting than with changes in spikelet number or individual grain weight. Previous studies have shown that flooding during the jointing to booting period can reduce tiller survival and interfere with panicle development, thereby limiting reproductive sink formation [16,17]. Flooding can also impair photosynthetic and hydraulic functions, which restricts assimilate supply during recovery and reproductive development [15,19,26]. The higher effective panicle number and seed setting rate under CI were therefore consistent with better maintenance of the yield components that are particularly sensitive during the transition to reproductive growth.

The response of yield components under CI was also consistent with the observed changes in canopy function and biomass allocation. Compared with CF, CI restrained excessive late tillering and canopy expansion while maintaining a higher net photosynthetic rate, greater root dry matter, and a higher root-to-shoot ratio. Maintenance of root biomass can support water and nutrient acquisition, whereas sustained photosynthetic activity provides assimilates for panicle development and grain formation [15,19,21]. The concurrence of these responses suggests that improved coordination between carbon assimilation and belowground biomass maintenance contributed to reproductive development after flooding. Accordingly, the lower yield loss under CI was associated with better maintenance of effective panicle number and seed setting rate. These findings extend previous research on rice flooding at the jointing stage by showing that antecedent irrigation management can influence how flooding responses are translated into final grain yield.

### 3.4. Practical Implications and Future Research

Water-saving irrigation can reduce irrigation inputs while maintaining rice yield and water productivity under appropriate management [11,20,29,30]. The present pot experiment provided consistent control of flooding depth and duration, allowing the influence of the antecedent water regime on rice responses to the jointing stage flooding to be clearly compared. Under these conditions, CI moderated excessive vegetative growth, maintained photosynthetic activity and root biomass, and was associated with lower yield loss than CF. These findings indicate that water management before flooding may contribute to yield stability when short periods of flooding occur during the jointing stage.

Application at the field scale requires coordination between irrigation and drainage. Spatial variation in soil texture, field elevation, infiltration, and drainage capacity can produce uneven soil moisture and surface water depth within the same field. Consequently, CI should be supported by representative soil moisture monitoring, appropriate field leveling, and timely drainage. Irrigation decisions may also be improved by incorporating rainfall forecasts and dynamically adjusting the capacity of paddy fields to store rainfall [10,12,13,24]. CI should therefore complement, rather than replace, effective drainage management, particularly in areas exposed to intense rainfall.

The pot approach reduced environmental variation and strengthened comparisons among treatments, although field hydrological processes are more complex because they involve runoff, lateral seepage, spatially variable infiltration, and gradual water recession. Wuyoudao 4, the cultivar used in this study, is a major japonica rice cultivar in the experimental region and is therefore relevant to local production. Future studies could include cultivars with contrasting plant architecture and flooding tolerance. Field experiments across different soil conditions, rainfall patterns, and drainage capacities are also needed to evaluate the effects of CI on grain yield, water productivity, and operational feasibility under commercial production conditions.

## 4. Materials and Methods

### 4.1. Experimental Site

**Figure 6:**
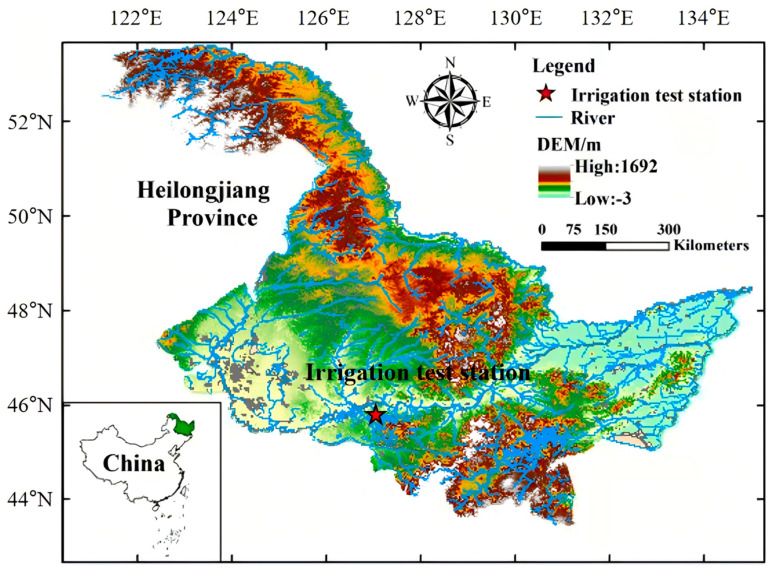
Study area.

The experiments were carried out during the 2023 and 2024 rice growing seasons at the Heilongjiang Provincial Water Conservancy Science and Technology Experimental Research Center in Harbin, Northeast China (45°43′09″ N, 126°36′35″ E; 137.0 m above sea level) (Figure 6). The site is located in a mid-temperate continental monsoon region, with pronounced seasonal variation and highly concentrated summer rainfall.

The long-term mean annual air temperature ranges from −4 to 5 °C, and the frost free period lasts approximately 130 to 140 days. Annual precipitation averages 400 to 650 mm, with nearly 70% occurring between July and September. The experimental area lies within the typical black soil region of Northeast China. The soil profile (0–80 cm) is classified as sandy loam. In the plow layer, the available nitrogen and potassium (K_2_O) contents are 154.4 mg·kg^−1^ and 376.8 mg·kg^−1^, respectively, the soil organic matter content is 25.07 g· kg^−1^, and the soil pH is 7.27. The mean field capacity of the 0–100 cm soil layer is 28.57%.

### 4.2. Experimental Design

A japonica rice cultivar, Wuyoudao 4 (Heishendao 2009005), was used in 2023 and 2024. The cultivar has a growth duration of approximately 147 days and an accumulated temperature requirement of about 2800 °C (≥10 °C).

The experiment comprised 14 treatment combinations under two irrigation regimes: controlled irrigation (CI) and conventional flooding irrigation (CF). Within each irrigation regime, three flooding depths, 1/3 of plant height (S), 2/3 of plant height (M), and full plant height corresponding to complete submergence (D), were combined with two flooding durations, 3 and 7 days, together with a non-flooded control (CK) (Table 1).

Under CI, a shallow water layer of 10–30 mm was maintained only during the seedling recovery stage after transplanting. Thereafter, irrigation was scheduled according to root-zone soil moisture thresholds, and no standing water was maintained on the soil surface. Under CF, a shallow water layer was maintained during most growth stages except the midseason drainage period, following local irrigation practices (Table 2).

**Figure 7:**
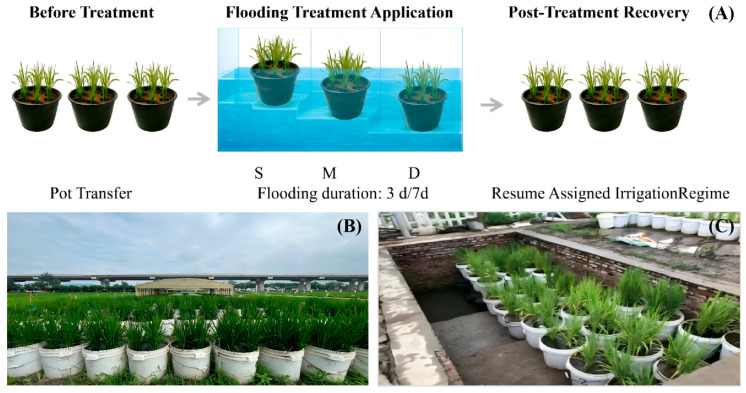
Experimental setup of the movable submergence method using pots. (**A**) Schematic diagram of the flooding treatment procedure. (**B**) Pot experiment at the study site. (**C**) Submergence tank during flooding treatment.

Flooding treatments were imposed at the beginning of the jointing stage using a movable submergence system (Figure 7). Plant height was measured immediately before flooding, and the target water depth was set relative to plant height in each experimental year. Pots were transferred to a submergence tank containing three water-depth sections corresponding to S, M, and D. Pots were randomly positioned within each section, and the target water levels were maintained throughout the flooding period.

For each treatment combination, six pots were established each year: three for growth and physiological measurements and final grain yield determination, and three for destructive biomass sampling. Thus, 84 pots were used each year. Each pot contained three rice hills and served as an independent experimental replicate. Tiller number, total leaf area, organ dry matter, effective panicle number, and grain yield were determined for the entire pot. Measurements on individual plants or leaves were averaged within each pot.

At the end of each flooding treatment, the pots were removed from the tank, and surface water was manually removed until the soil remained saturated without visible standing water. The pots were then returned to their original positions, and irrigation resumed according to the assigned regime. Treatment combinations were arranged in a randomized block design, and all other agronomic practices were identical across treatments.

### 4.3. Measurement Items and Methods

#### 4.3.1. Vegetative Growth Traits

Vegetative growth traits were measured on the first day after flooding termination. The tiller number was determined by counting all tillers from the three rice hills in each pot. Plant height was measured from the soil surface to the highest leaf tip, and measurements within each pot were averaged.

#### 4.3.2. Leaf Area and Photosynthetic Characteristics

Leaf area and net photosynthetic rate (Pn) were measured on the first day after flooding termination. The length (*L_i_*) and maximum width (*W_i_*) of each green leaf from the three rice hills in each pot were measured. Total leaf area (TLA) per pot was calculated as:

$$TLA=0.83\sum_{i=1}^{n}L_{i}W_{i}$$

where TLA is total leaf area (cm^2^·pot^−1^); *L_i_* and *W_i_* are the length and maximum width of the leaf (cm), respectively; n is the total number of green leaves in the three hills within each pot; and 0.83 is the shape coefficient.

Net photosynthetic rate (Pn) was measured on fully expanded upper leaves using a portable photosynthesis system (CI-340, CID Bio-Science, Inc., Camas, WA, USA). Measurements were conducted during midmorning hours to minimize diurnal variation.

#### 4.3.3. Biomass Accumulation and Allocation

On the first day after flooding termination, all plants in each biomass sampling pot were separated into roots, stems, and leaves. Samples were oven dried at 105 °C for 30 min and then at 80 °C to constant weight. Dry matter was measured using an electronic balance with a precision of 0.001 g. Aboveground dry matter was calculated as the sum of stem and leaf dry matter. The root-to-shoot ratio was calculated for each pot as root dry matter divided by aboveground dry matter.

#### 4.3.4. Grain Yield and Yield Components

At physiological maturity, all plants in each yield measurement pot were harvested. Effective panicle number was counted for the three rice hills in each pot. All productive panicles were threshed, and grain yield was determined for each pot. Spikelet number per panicle was calculated as the total number of spikelets divided by the effective panicle number. Seed setting rate was calculated as the percentage of filled grains relative to total spikelets. The 1000-grain weight was determined using filled grains harvested from each pot.

### 4.4. Statistical Analysis

Prior to data pooling, a preliminary analysis of variance (ANOVA) was performed to test the effects of year, treatment combination, and their interaction. Because the year × treatment combination interaction was not significant (*p* > 0.05), data from 2023 and 2024 were pooled for subsequent analyses.

All measured variables were analyzed using one-way ANOVA, with treatment combination treated as the fixed factor. The treatment combinations consisted of the two irrigation regimes, CI and CF, under seven flooding scenarios: CK, S3, S7, M3, M7, D3, and D7. When significant treatment effects were detected, mean comparisons were performed using Tukey’s honestly significant difference (HSD) test at *p* < 0.05.

Relative changes and yield-loss percentages were calculated using the corresponding non-flooded control within each irrigation regime as the reference. Results are presented as means ± standard error (SE) across two years, with three replicates per year (n = 6). Pearson correlation analysis was used to examine relationships among vegetative growth traits, photosynthetic characteristics, biomass allocation variables, yield components, and grain yield. Correlation patterns were visualized using a heatmap to facilitate interpretation. All statistical analyses were conducted using SPSS 19.0 (IBM Corp., Armonk, NY, USA), and figures were prepared using Origin 2026 (OriginLab Corp., Northampton, MA, USA).

## 5. Conclusions

Flooding at the jointing stage stimulated vegetative expansion but reduced net photosynthetic rate, root biomass, and grain yield. Under corresponding flooding conditions, CI moderated increases in tiller number, plant height, and total leaf area while maintaining higher net photosynthetic rates, greater root dry matter, and a higher root-to-shoot ratio than CF. CI also maintained a greater effective panicle number and a higher seed setting rate, and both traits were closely associated with the lower yield loss. The advantage of CI was linked to a more balanced response in shoot growth, canopy carbon assimilation, belowground biomass maintenance, and reproductive development. The soil water regime before flooding can therefore shape rice growth and yield formation during subsequent flooding at the jointing stage. In practice, controlled irrigation should be combined with timely drainage to reduce yield risk under short-term flooding. Field studies across cultivars, soil conditions, rainfall patterns, and drainage capacities are needed to evaluate its performance under commercial production conditions.

## Figures and Tables

**Table 1:** Experimental treatments under different irrigation regimes and flooding conditions.

Irrigation Regime	Treatment Code	Flooding Depth *	Flooding Duration
CI	CI-S3	1/3 plant height	3 d
CI	CI-S7	1/3 plant height	7 d
CI	CI-M3	2/3 plant height	3 d
CI	CI-M7	2/3 plant height	7 d
CI	CI-D3	Full plant height	3 d
CI	CI-D7	Full plant height	7 d
CI	CI-CK	No flooding	–
CF	CF-S3	1/3 plant height	3 d
CF	CF-S7	1/3 plant height	7 d
CF	CF-M3	2/3 plant height	3 d
CF	CF-M7	2/3 plant height	7 d
CF	CF-D3	Full plant height	3 d
CF	CF-D7	Full plant height	7 d
CF	CF-CK	No flooding	–

Notes: * Flooding depth was defined relative to plant height at the time of treatment. Flooding treatments were imposed at the jointing stage using a movable submergence method with pots.

**Table 2:** Upper and lower irrigation thresholds at different rice growth stages under controlled irrigation and conventional flooding irrigation.

Irrigation Regime	Threshold	Regreening Stage	Tillering Stage	Jointing Stage	Heading Stage	Milk Stage	Yellow Maturity Stage
CI	Upper limit (mm)	30	20	20	0	20	-
	Lower limit (%)	80	85	85	60	85	-
CF	Upper limit (mm)	30	50	50	50	50	-
	Lower limit (mm)	10	30	30	30	30	-

Notes: (1) Values expressed as percentages (%) indicate the proportion of soil saturated water content, whereas values expressed in millimeters (mm) represent surface water depth. (2) The lower soil moisture thresholds at each growth stage represent the average water content of the root zone soil layer.

## Data Availability

Data supporting the findings of this study are available on request from the corresponding author.
